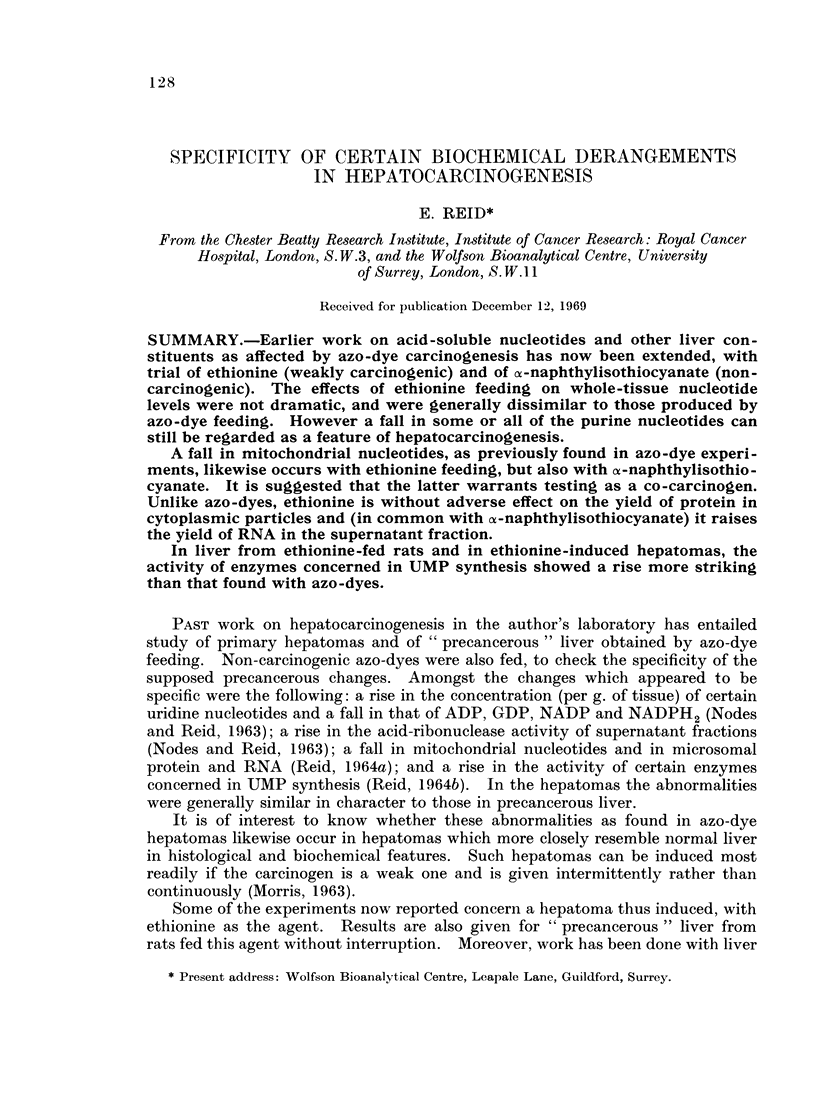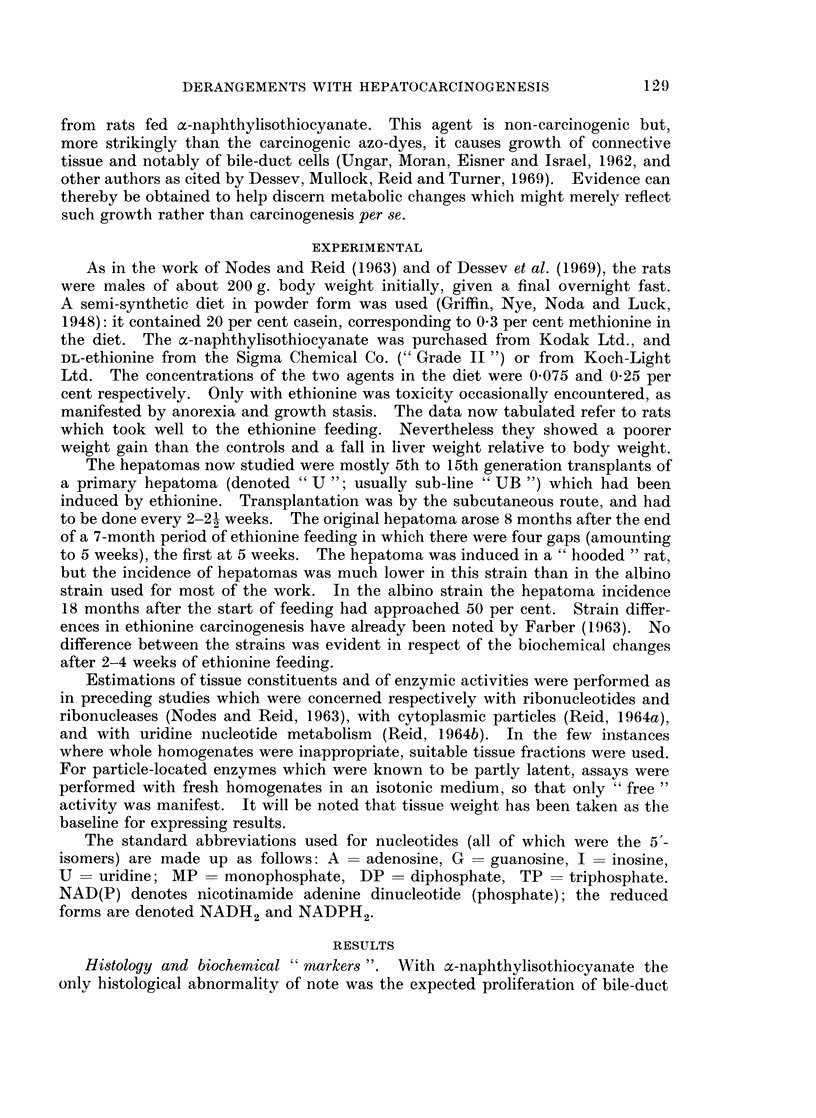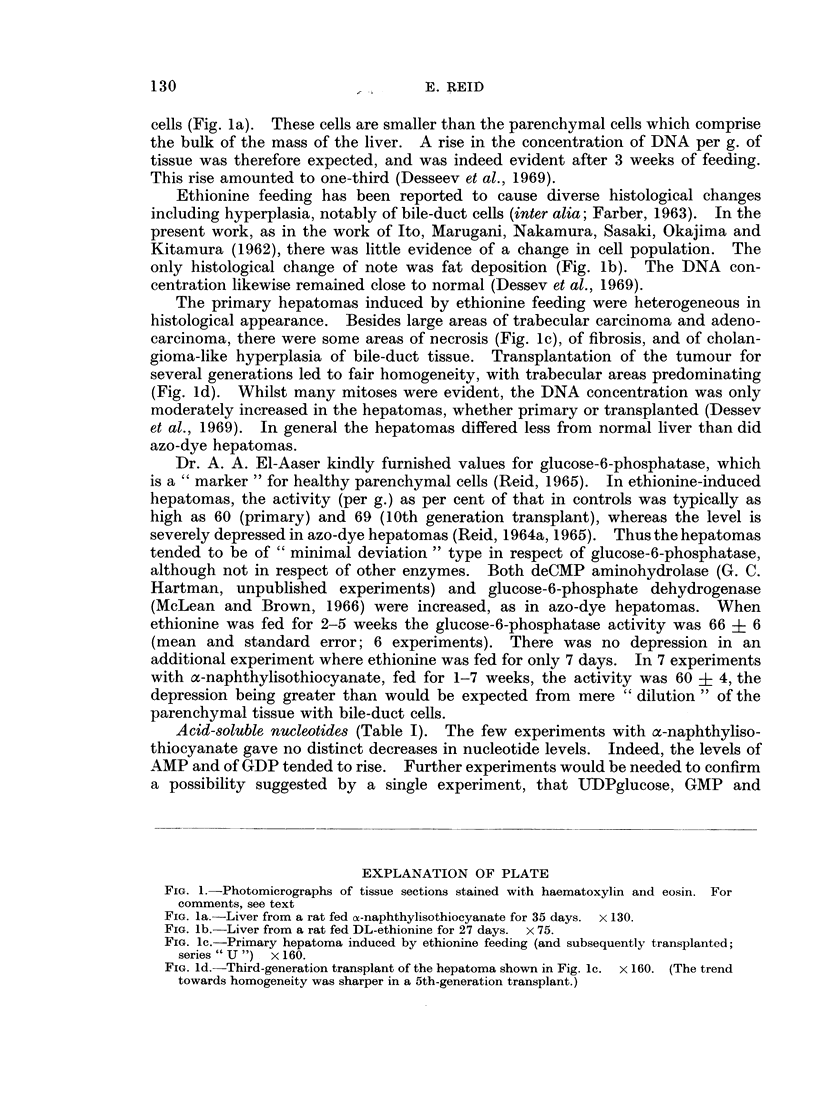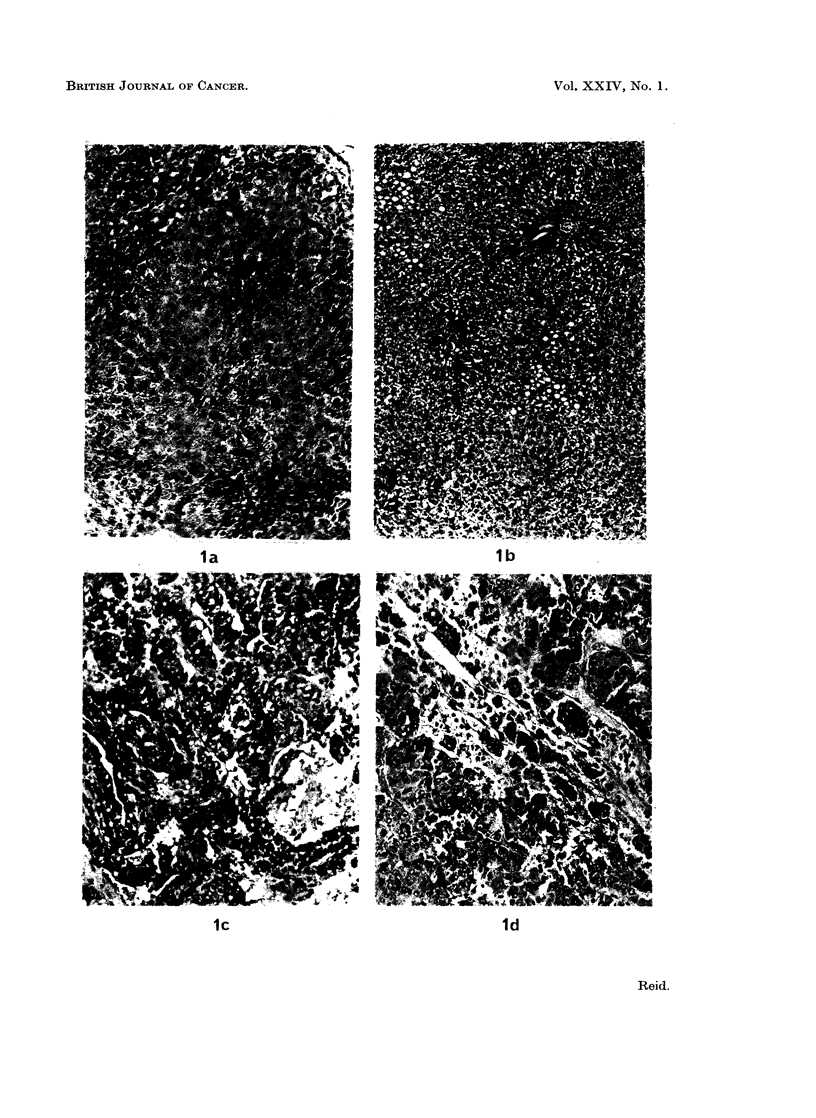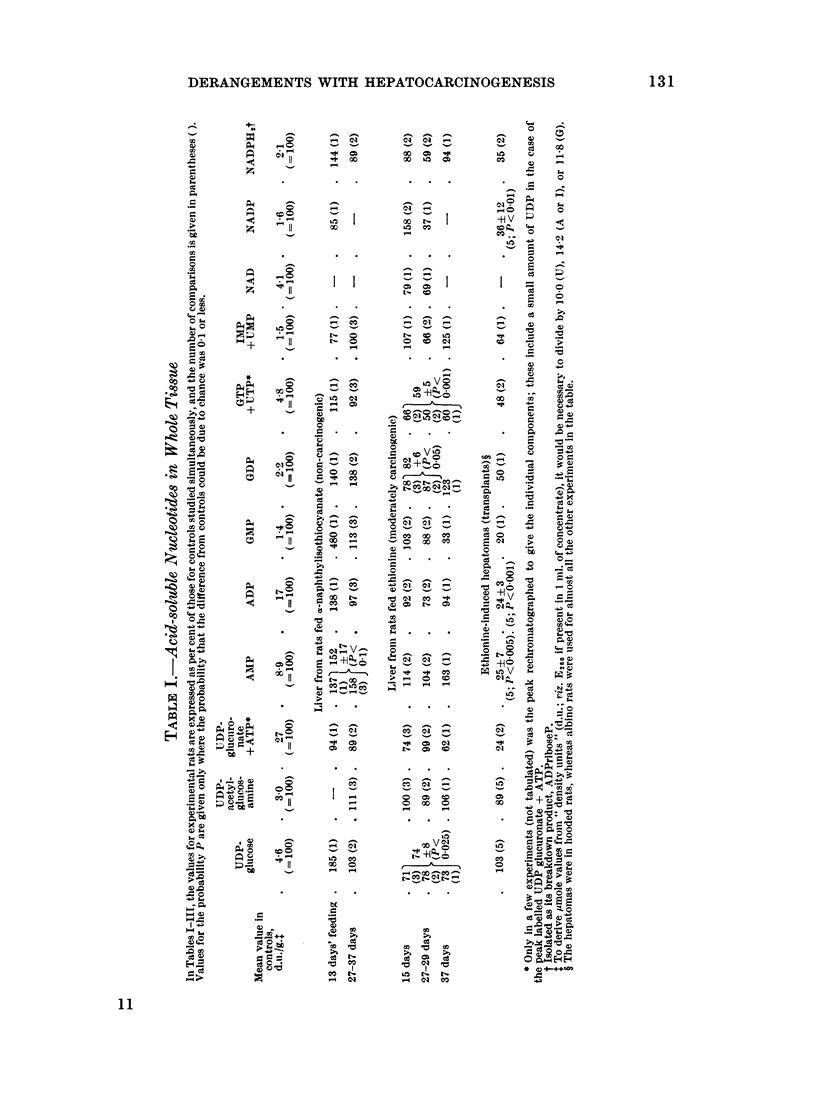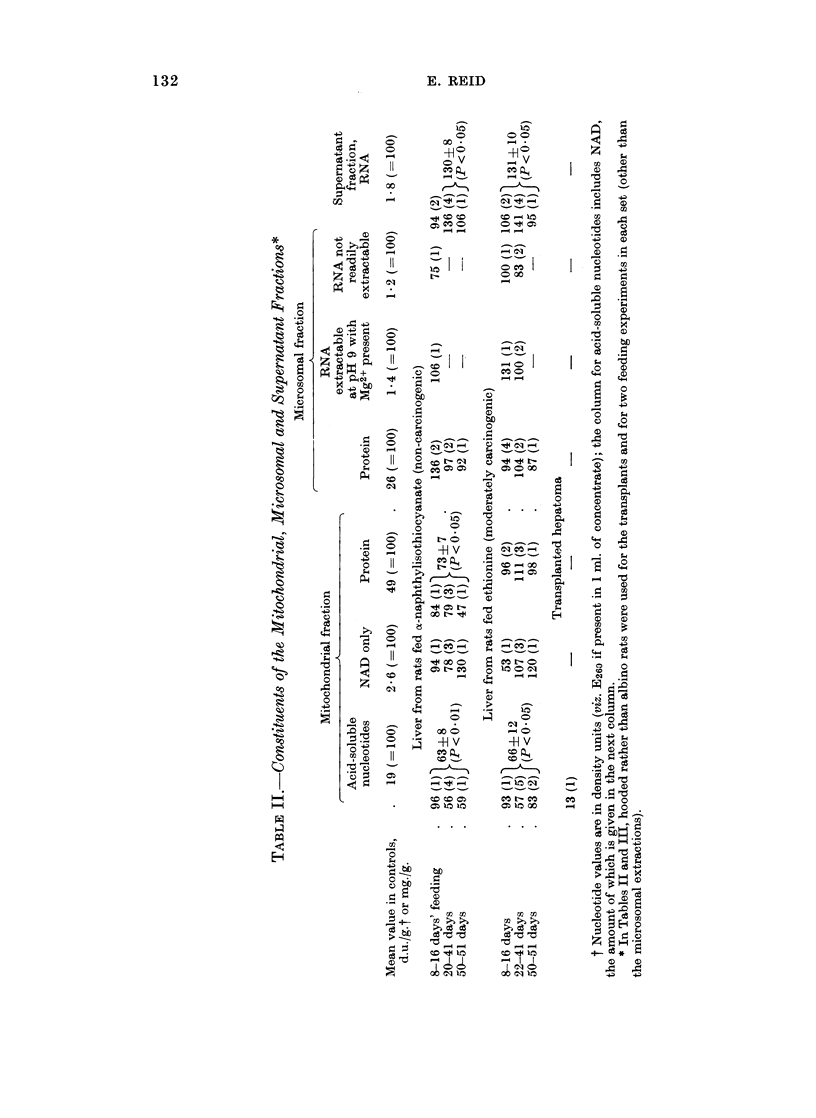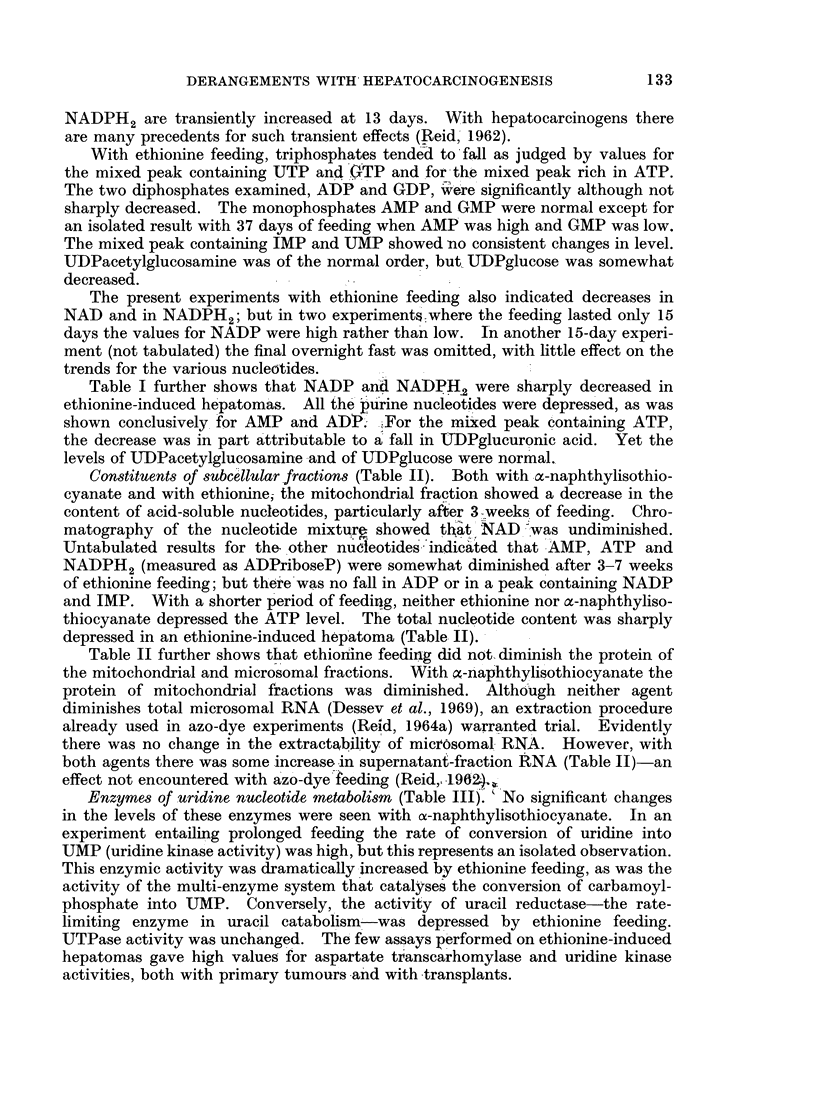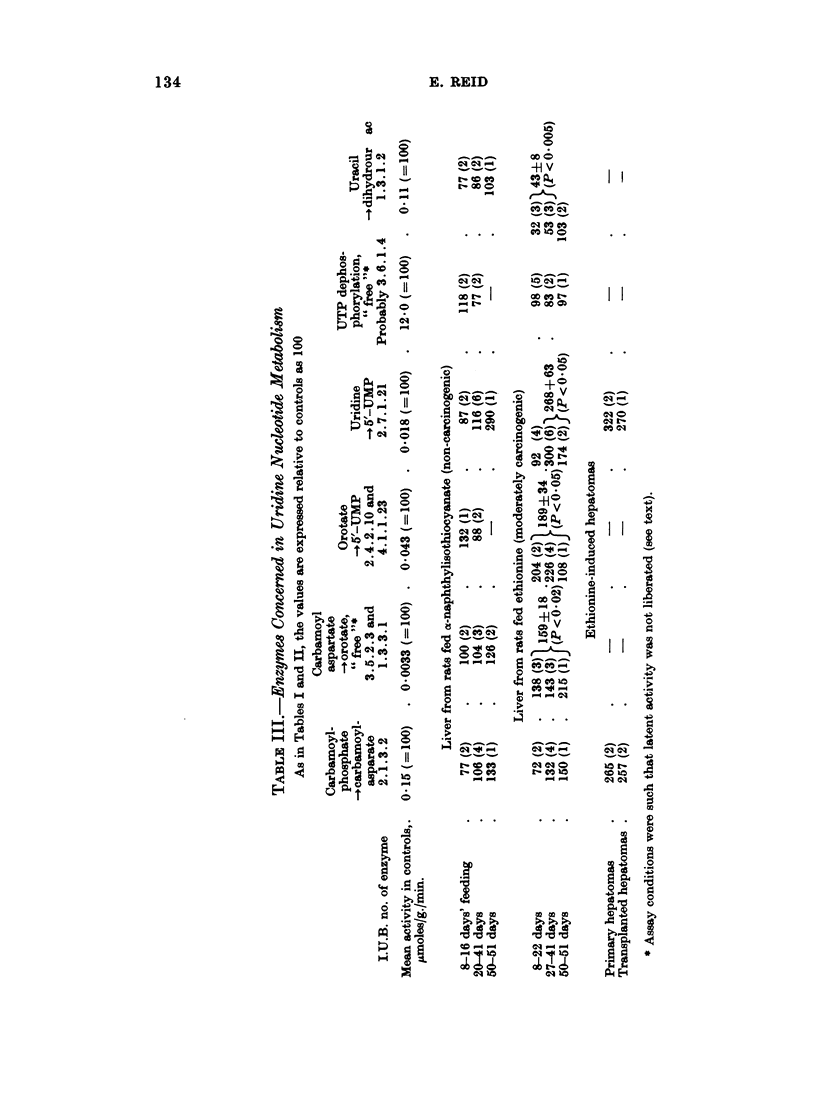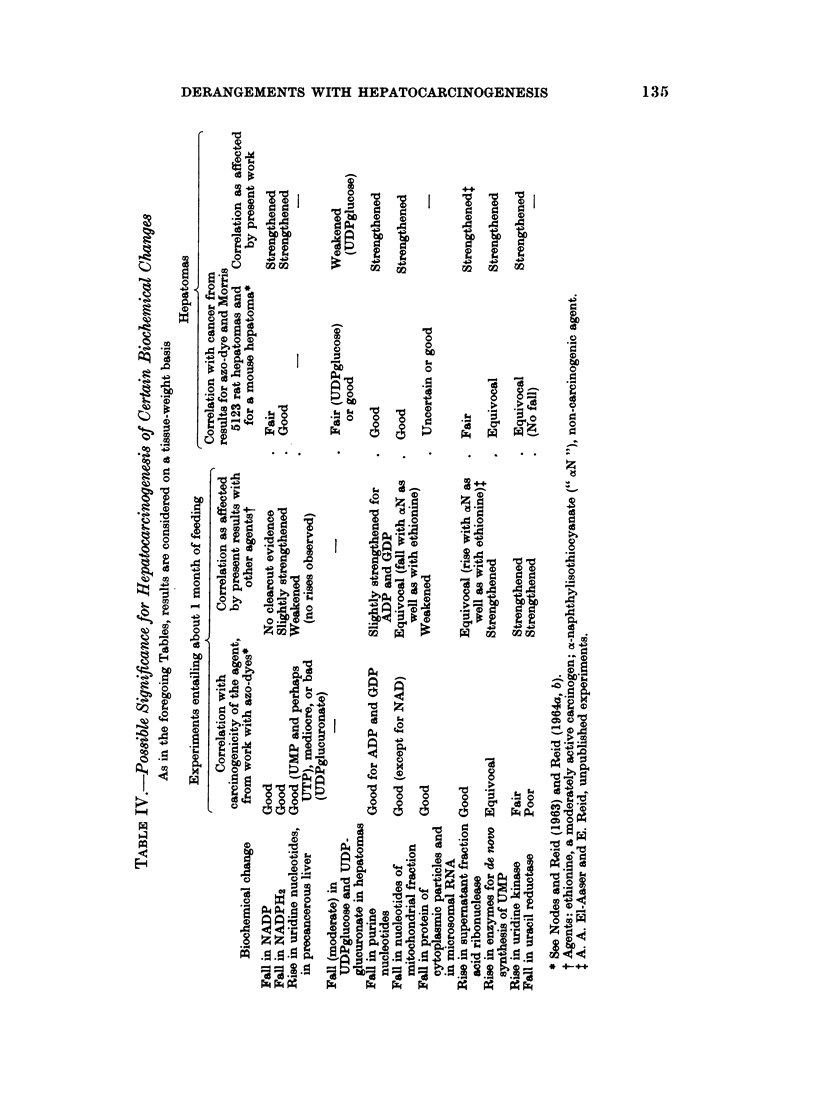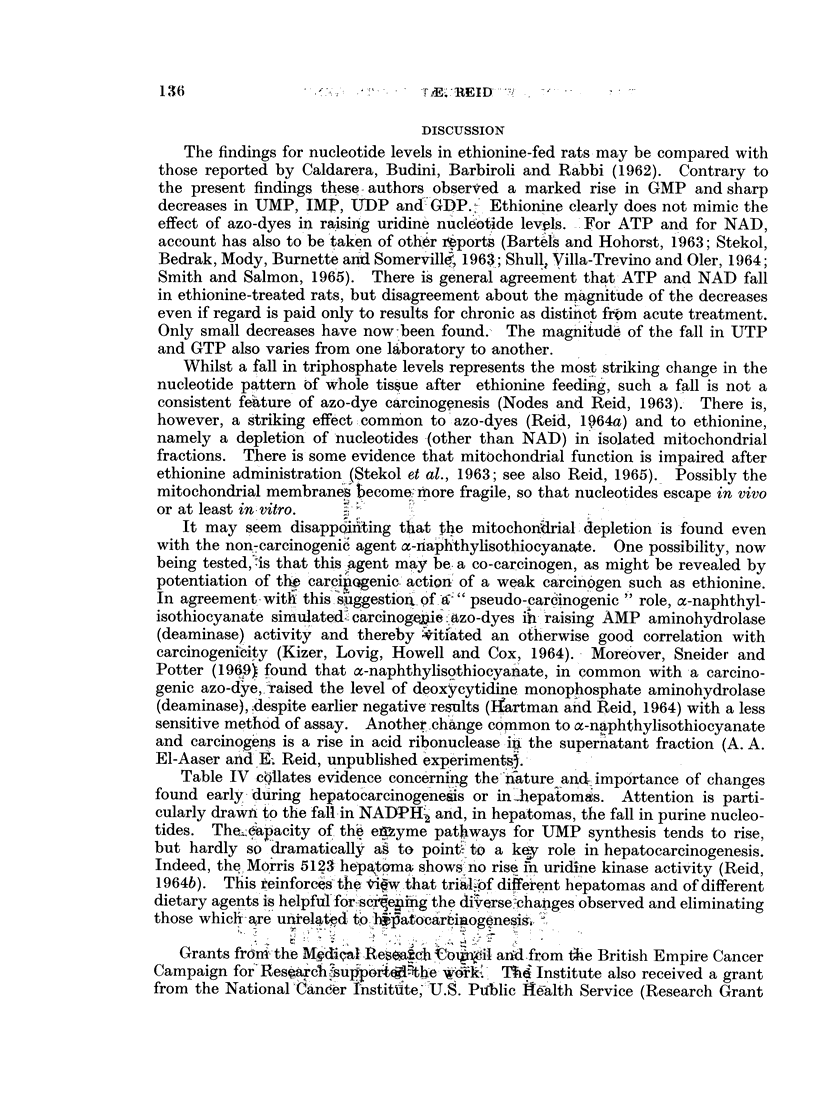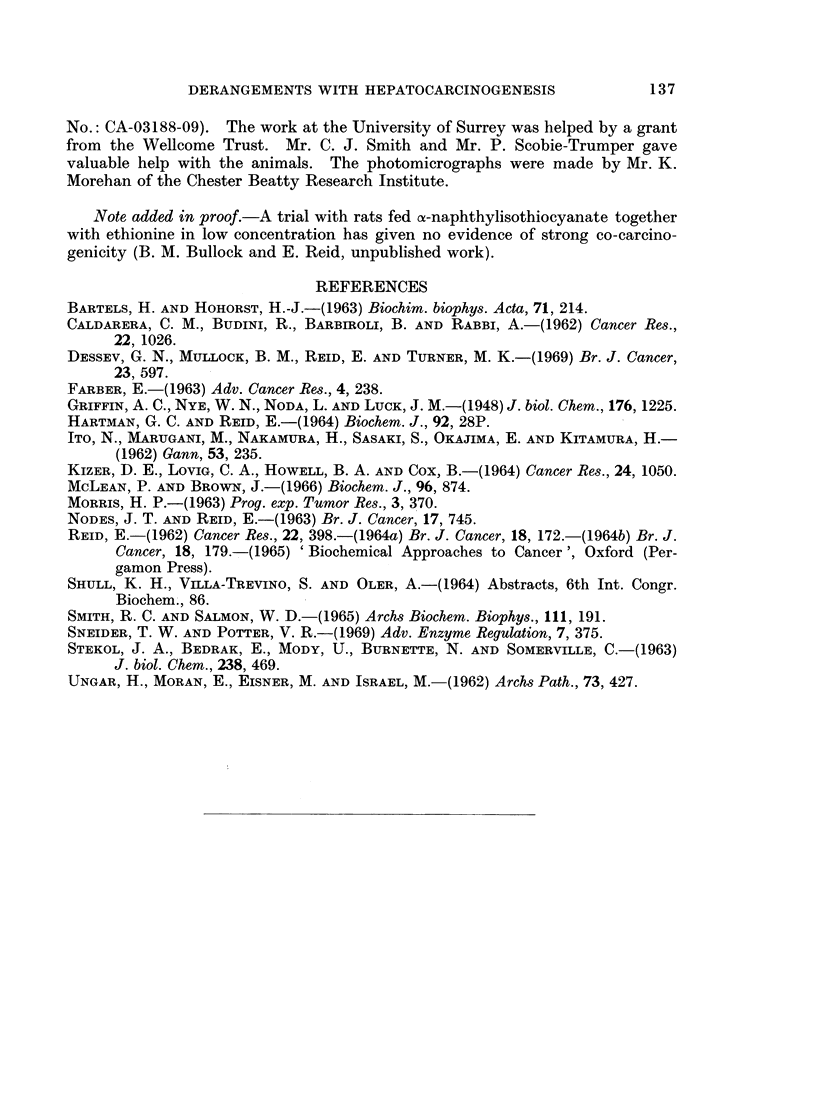# Specificity of Certain Biochemical Derangements in Hepatocarcinogenesis

**DOI:** 10.1038/bjc.1970.16

**Published:** 1970-03

**Authors:** E. Reid

## Abstract

**Images:**


### SPECIFICITY OF CERTAIN BIOCHEMICAL DERANGEMENTS IN HEPATOCARCINOGENESIS

E. REID*

*From the Chester Beatty Research Institute, Institute of Cancer Research: Royal Cancer Hospital, London, S.W.3, and the Wolfson Bioanalytical Centre, University of Surrey, London, S.W.11*

Received for publication December 12, 1969

**SUMMARY.—Earlier work on acid-soluble nucleotides and other liver constituents as affected by azo-dye carcinogenesis has now been extended, with trial of ethionine (weakly carcinogenic) and of α-naphthylisothiocyanate (non-carcinogenic). The effects of ethionine feeding on whole-tissue nucleotide levels were not dramatic, and were generally dissimilar to those produced by azo-dye feeding. However a fall in some or all of the purine nucleotides can still be regarded as a feature of hepatocarcinogenesis.**

**A fall in mitochondrial nucleotides, as previously found in azo-dye experiments, likewise occurs with ethionine feeding, but also with α-naphthylisothiocyanate. It is suggested that the latter warrants testing as a co-carcinogen. Unlike azo-dyes, ethionine is without adverse effect on the yield of protein in cytoplasmic particles and (in common with α-naphthylisothiocyanate) it raises the yield of RNA in the supernatant fraction.**

**In liver from ethionine-fed rats and in ethionine-induced hepatomas, the activity of enzymes concerned in UMP synthesis showed a rise more striking than that found with azo-dyes.**

PAST work on hepatocarcinogenesis in the author's laboratory has entailed study of primary hepatomas and of " precancerous " liver obtained by azo-dye feeding. Non-carcinogenic azo-dyes were also fed, to check the specificity of the supposed precancerous changes. Amongst the changes which appeared to be specific were the following: a rise in the concentration (per g. of tissue) of certain uridine nucleotides and a fall in that of ADP, GDP, NADP and $NADPH_2$ (Nodes and Reid, 1963); a rise in the acid-ribonuclease activity of supernatant fractions (Nodes and Reid, 1963); a fall in mitochondrial nucleotides and in microsomal protein and RNA (Reid, 1964*a*); and a rise in the activity of certain enzymes concerned in UMP synthesis (Reid, 1964*b*). In the hepatomas the abnormalities were generally similar in character to those in precancerous liver.

It is of interest to know whether these abnormalities as found in azo-dye hepatomas likewise occur in hepatomas which more closely resemble normal liver in histological and biochemical features. Such hepatomas can be induced most readily if the carcinogen is a weak one and is given intermittently rather than continuously (Morris, 1963).

Some of the experiments now reported concern a hepatoma thus induced, with ethionine as the agent. Results are also given for " precancerous " liver from rats fed this agent without interruption. Moreover, work has been done with liver

* Present address: Wolfson Bioanalytical Centre, Leapale Lane, Guildford, Surrey.

from rats fed α-naphthylisothiocyanate. This agent is non-carcinogenic but, more strikingly than the carcinogenic azo-dyes, it causes growth of connective tissue and notably of bile-duct cells (Ungar, Moran, Eisner and Israel, 1962, and other authors as cited by Dessev, Mullock, Reid and Turner, 1969). Evidence can thereby be obtained to help discern metabolic changes which might merely reflect such growth rather than carcinogenesis *per se.*

### EXPERIMENTAL

As in the work of Nodes and Reid (1963) and of Dessev *et al.* (1969), the rats were males of about 200 g. body weight initially, given a final overnight fast. A semi-synthetic diet in powder form was used (Griffin, Nye, Noda and Luck, 1948): it contained 20 per cent casein, corresponding to 0·3 per cent methionine in the diet. The α-naphthylisothiocyanate was purchased from Kodak Ltd., and DL-ethionine from the Sigma Chemical Co. ("Grade II") or from Koch-Light Ltd. The concentrations of the two agents in the diet were 0·075 and 0·25 per cent respectively. Only with ethionine was toxicity occasionally encountered, as manifested by anorexia and growth stasis. The data now tabulated refer to rats which took well to the ethionine feeding. Nevertheless they showed a poorer weight gain than the controls and a fall in liver weight relative to body weight.

The hepatomas now studied were mostly 5th to 15th generation transplants of a primary hepatoma (denoted "U"; usually sub-line "UB") which had been induced by ethionine. Transplantation was by the subcutaneous route, and had to be done every 2–2½ weeks. The original hepatoma arose 8 months after the end of a 7-month period of ethionine feeding in which there were four gaps (amounting to 5 weeks), the first at 5 weeks. The hepatoma was induced in a "hooded" rat, but the incidence of hepatomas was much lower in this strain than in the albino strain used for most of the work. In the albino strain the hepatoma incidence 18 months after the start of feeding had approached 50 per cent. Strain differences in ethionine carcinogenesis have already been noted by Farber (1963). No difference between the strains was evident in respect of the biochemical changes after 2–4 weeks of ethionine feeding.

Estimations of tissue constituents and of enzymic activities were performed as in preceding studies which were concerned respectively with ribonucleotides and ribonucleases (Nodes and Reid, 1963), with cytoplasmic particles (Reid, 1964*a*), and with uridine nucleotide metabolism (Reid, 1964*b*). In the few instances where whole homogenates were inappropriate, suitable tissue fractions were used. For particle-located enzymes which were known to be partly latent, assays were performed with fresh homogenates in an isotonic medium, so that only "free" activity was manifest. It will be noted that tissue weight has been taken as the baseline for expressing results.

The standard abbreviations used for nucleotides (all of which were the 5′-isomers) are made up as follows: A = adenosine, G = guanosine, I = inosine, U = uridine; MP = monophosphate, DP = diphosphate, TP = triphosphate. NAD(P) denotes nicotinamide adenine dinucleotide (phosphate); the reduced forms are denoted $NADH_2$ and $NADPH_2$.

### RESULTS

*Histology and biochemical "markers".* With α-naphthylisothiocyanate the only histological abnormality of note was the expected proliferation of bile-duct cells (Fig. 1a). These cells are smaller than the parenchymal cells which comprise the bulk of the mass of the liver. A rise in the concentration of DNA per g. of tissue was therefore expected, and was indeed evident after 3 weeks of feeding. This rise amounted to one-third (Desseev *et al.*, 1969).

Ethionine feeding has been reported to cause diverse histological changes including hyperplasia, notably of bile-duct cells (*inter alia*; Farber, 1963). In the present work, as in the work of Ito, Marugani, Nakamura, Sasaki, Okajima and Kitamura (1962), there was little evidence of a change in cell population. The only histological change of note was fat deposition (Fig. 1b). The DNA concentration likewise remained close to normal (Dessev *et al.*, 1969).

The primary hepatomas induced by ethionine feeding were heterogeneous in histological appearance. Besides large areas of trabecular carcinoma and adenocarcinoma, there were some areas of necrosis (Fig. 1c), of fibrosis, and of cholangioma-like hyperplasia of bile-duct tissue. Transplantation of the tumour for several generations led to fair homogeneity, with trabecular areas predominating (Fig. 1d). Whilst many mitoses were evident, the DNA concentration was only moderately increased in the hepatomas, whether primary or transplanted (Dessev *et al.*, 1969). In general the hepatomas differed less from normal liver than did azo-dye hepatomas.

Dr. A. A. El-Aaser kindly furnished values for glucose-6-phosphatase, which is a " marker " for healthy parenchymal cells (Reid, 1965). In ethionine-induced hepatomas, the activity (per g.) as per cent of that in controls was typically as high as 60 (primary) and 69 (10th generation transplant), whereas the level is severely depressed in azo-dye hepatomas (Reid, 1964a, 1965). Thus the hepatomas tended to be of " minimal deviation " type in respect of glucose-6-phosphatase, although not in respect of other enzymes. Both deCMP aminohydrolase (G. C. Hartman, unpublished experiments) and glucose-6-phosphate dehydrogenase (McLean and Brown, 1966) were increased, as in azo-dye hepatomas. When ethionine was fed for 2–5 weeks the glucose-6-phosphatase activity was $66 \pm 6$ (mean and standard error; 6 experiments). There was no depression in an additional experiment where ethionine was fed for only 7 days. In 7 experiments with $\alpha$-naphthylisothiocyanate, fed for 1–7 weeks, the activity was $60 \pm 4$, the depression being greater than would be expected from mere " dilution " of the parenchymal tissue with bile-duct cells.

*Acid-soluble nucleotides* (Table I). The few experiments with $\alpha$-naphthylisothiocyanate gave no distinct decreases in nucleotide levels. Indeed, the levels of AMP and of GDP tended to rise. Further experiments would be needed to confirm a possibility suggested by a single experiment, that UDPglucose, GMP and

---

EXPLANATION OF PLATE

FIG. 1.—Photomicrographs of tissue sections stained with haematoxylin and eosin. For comments, see text

FIG. 1a.—Liver from a rat fed $\alpha$-naphthylisothiocyanate for 35 days. ×130.

FIG. 1b.—Liver from a rat fed DL-ethionine for 27 days. ×75.

FIG. 1c.—Primary hepatoma induced by ethionine feeding (and subsequently transplanted; series " U ") ×160.

FIG. 1d.—Third-generation transplant of the hepatoma shown in Fig. 1c. ×160. (The trend towards homogeneity was sharper in a 5th-generation transplant.)

$NADPH_2$ are transiently increased at 13 days. With hepatocarcinogens there are many precedents for such transient effects (Reid, 1962).

With ethionine feeding, triphosphates tended to fall as judged by values for the mixed peak containing UTP and GTP and for the mixed peak rich in ATP. The two diphosphates examined, ADP and GDP, were significantly although not sharply decreased. The monophosphates AMP and GMP were normal except for an isolated result with 37 days of feeding when AMP was high and GMP was low. The mixed peak containing IMP and UMP showed no consistent changes in level. UDPacetylglucosamine was of the normal order, but UDPglucose was somewhat decreased.

The present experiments with ethionine feeding also indicated decreases in NAD and in $NADPH_2$; but in two experiments where the feeding lasted only 15 days the values for NADP were high rather than low. In another 15-day experiment (not tabulated) the final overnight fast was omitted, with little effect on the trends for the various nucleotides.

Table I further shows that NADP and $NADPH_2$ were sharply decreased in ethionine-induced hepatomas. All the purine nucleotides were depressed, as was shown conclusively for AMP and ADP. For the mixed peak containing ATP, the decrease was in part attributable to a fall in UDPglucuronic acid. Yet the levels of UDPacetylglucosamine and of UDPglucose were normal.

*Constituents of subcellular fractions* (Table II). Both with $\alpha$-naphthylisothiocyanate and with ethionine, the mitochondrial fraction showed a decrease in the content of acid-soluble nucleotides, particularly after 3 weeks of feeding. Chromatography of the nucleotide mixture showed that NAD was undiminished. Untabulated results for the other nucleotides indicated that AMP, ATP and $NADPH_2$ (measured as ADPriboseP) were somewhat diminished after 3–7 weeks of ethionine feeding; but there was no fall in ADP or in a peak containing NADP and IMP. With a shorter period of feeding, neither ethionine nor $\alpha$-naphthylisothiocyanate depressed the ATP level. The total nucleotide content was sharply depressed in an ethionine-induced hepatoma (Table II).

Table II further shows that ethionine feeding did not diminish the protein of the mitochondrial and microsomal fractions. With $\alpha$-naphthylisothiocyanate the protein of mitochondrial fractions was diminished. Although neither agent diminishes total microsomal RNA (Dessev *et al.*, 1969), an extraction procedure already used in azo-dye experiments (Reid, 1964a) warranted trial. Evidently there was no change in the extractability of microsomal RNA. However, with both agents there was some increase in supernatant-fraction RNA (Table II)—an effect not encountered with azo-dye feeding (Reid, 1962).

*Enzymes of uridine nucleotide metabolism* (Table III). No significant changes in the levels of these enzymes were seen with $\alpha$-naphthylisothiocyanate. In an experiment entailing prolonged feeding the rate of conversion of uridine into UMP (uridine kinase activity) was high, but this represents an isolated observation. This enzymic activity was dramatically increased by ethionine feeding, as was the activity of the multi-enzyme system that catalyses the conversion of carbamoylphosphate into UMP. Conversely, the activity of uracil reductase—the rate-limiting enzyme in uracil catabolism—was depressed by ethionine feeding. UTPase activity was unchanged. The few assays performed on ethionine-induced hepatomas gave high values for aspartate transcarhomylase and uridine kinase activities, both with primary tumours and with transplants.

‡ A. A. El-Aaser and E. Reid, unpublished experiments.

### DISCUSSION

The findings for nucleotide levels in ethionine-fed rats may be compared with those reported by Caldarera, Budini, Barbiroli and Rabbi (1962). Contrary to the present findings these authors observed a marked rise in GMP and sharp decreases in UMP, IMP, UDP and GDP. Ethionine clearly does not mimic the effect of azo-dyes in raising uridine nucleotide levels. For ATP and for NAD, account has also to be taken of other reports (Bartels and Hohorst, 1963; Stekol, Bedrak, Mody, Burnette and Somerville, 1963; Shull, Villa-Trevino and Oler, 1964; Smith and Salmon, 1965). There is general agreement that ATP and NAD fall in ethionine-treated rats, but disagreement about the magnitude of the decreases even if regard is paid only to results for chronic as distinct from acute treatment. Only small decreases have now been found. The magnitude of the fall in UTP and GTP also varies from one laboratory to another.

Whilst a fall in triphosphate levels represents the most striking change in the nucleotide pattern of whole tissue after ethionine feeding, such a fall is not a consistent feature of azo-dye carcinogenesis (Nodes and Reid, 1963). There is, however, a striking effect common to azo-dyes (Reid, 1964*a*) and to ethionine, namely a depletion of nucleotides (other than NAD) in isolated mitochondrial fractions. There is some evidence that mitochondrial function is impaired after ethionine administration (Stekol *et al.*, 1963; see also Reid, 1965). Possibly the mitochondrial membranes become more fragile, so that nucleotides escape *in vivo* or at least *in vitro*.

It may seem disappointing that the mitochondrial depletion is found even with the non-carcinogenic agent α-naphthylisothiocyanate. One possibility, now being tested, is that this agent may be a co-carcinogen, as might be revealed by potentiation of the carcinogenic action of a weak carcinogen such as ethionine. In agreement with this suggestion of a " pseudo-carcinogenic " role, α-naphthylisothiocyanate simulated carcinogenic azo-dyes in raising AMP aminohydrolase (deaminase) activity and thereby vitiated an otherwise good correlation with carcinogenicity (Kizer, Lovig, Howell and Cox, 1964). Moreover, Sneider and Potter (1969) found that α-naphthylisothiocyanate, in common with a carcinogenic azo-dye, raised the level of deoxycytidine monophosphate aminohydrolase (deaminase), despite earlier negative results (Hartman and Reid, 1964) with a less sensitive method of assay. Another change common to α-naphthylisothiocyanate and carcinogens is a rise in acid ribonuclease in the supernatant fraction (A. A. El-Aaser and E. Reid, unpublished experiments).

Table IV collates evidence concerning the nature and importance of changes found early during hepatocarcinogenesis or in hepatomas. Attention is particularly drawn to the fall in $NADPH_2$ and, in hepatomas, the fall in purine nucleotides. The capacity of the enzyme pathways for UMP synthesis tends to rise, but hardly so dramatically as to point to a key role in hepatocarcinogenesis. Indeed, the Morris 5123 hepatoma shows no rise in uridine kinase activity (Reid, 1964*b*). This reinforces the view that trial of different hepatomas and of different dietary agents is helpful for screening the diverse changes observed and eliminating those which are unrelated to hepatocarcinogenesis.

Grants from the Medical Research Council and from the British Empire Cancer Campaign for Research supported the work. The Institute also received a grant from the National Cancer Institute, U.S. Public Health Service (Research Grant

No.: CA-03188-09). The work at the University of Surrey was helped by a grant from the Wellcome Trust. Mr. C. J. Smith and Mr. P. Scobie-Trumper gave valuable help with the animals. The photomicrographs were made by Mr. K. Morehan of the Chester Beatty Research Institute.

*Note added in proof.*—A trial with rats fed α-naphthylisothiocyanate together with ethionine in low concentration has given no evidence of strong co-carcinogenicity (B. M. Bullock and E. Reid, unpublished work).

### REFERENCES

Bartels, H. and Hohorst, H.-J.—(1963) *Biochim. biophys. Acta*, **71**, 214.

Caldarera, C. M., Budini, R., Barbiroli, B. and Rabbi, A.—(1962) *Cancer Res.*, **22**, 1026.

Dessev, G. N., Mullock, B. M., Reid, E. and Turner, M. K.—(1969) *Br. J. Cancer*, **23**, 597.

Farber, E.—(1963) *Adv. Cancer Res.*, **4**, 238.

Griffin, A. C., Nye, W. N., Noda, L. and Luck, J. M.—(1948) *J. biol. Chem.*, **176**, 1225.

Hartman, G. C. and Reid, E.—(1964) *Biochem. J.*, **92**, 28P.

Ito, N., Marugani, M., Nakamura, H., Sasaki, S., Okajima, E. and Kitamura, H.—(1962) *Gann*, **53**, 235.

Kizer, D. E., Lovig, C. A., Howell, B. A. and Cox, B.—(1964) *Cancer Res.*, **24**, 1050.

McLean, P. and Brown, J.—(1966) *Biochem. J.*, **96**, 874.

Morris, H. P.—(1963) *Prog. exp. Tumor Res.*, **3**, 370.

Nodes, J. T. and Reid, E.—(1963) *Br. J. Cancer*, **17**, 745.

Reid, E.—(1962) *Cancer Res.*, **22**, 398.—(1964*a*) *Br. J. Cancer*, **18**, 172.—(1964*b*) *Br. J. Cancer*, **18**, 179.—(1965) ' Biochemical Approaches to Cancer ', Oxford (Pergamon Press).

Shull, K. H., Villa-Trevino, S. and Oler, A.—(1964) Abstracts, 6th Int. Congr. Biochem., 86.

Smith, R. C. and Salmon, W. D.—(1965) *Archs Biochem. Biophys.*, **111**, 191.

Sneider, T. W. and Potter, V. R.—(1969) *Adv. Enzyme Regulation*, **7**, 375.

Stekol, J. A., Bedrak, E., Mody, U., Burnette, N. and Somerville, C.—(1963) *J. biol. Chem.*, **238**, 469.

Ungar, H., Moran, E., Eisner, M. and Israel, M.—(1962) *Archs Path.*, **73**, 427.